# Article review: Brazilin as potential anticancer agent

**DOI:** 10.3389/fphar.2024.1355533

**Published:** 2024-03-07

**Authors:** Callista Najla Raptania, Syifa Zakia, Alistia Ilmiah Fahira, Riezki Amalia

**Affiliations:** ^1^ Department of Pharmacology and Clinical Pharmacy, Faculty of Pharmacy, Universitas Padjadjaran, Bandung, Indonesia; ^2^ Laboratory of Cell and Molecular Biology, Faculty of Pharmacy, Universitas Padjadjaran, Bandung, Indonesia; ^3^ Center of Excellence in Pharmaceutical Care Innovation, Universitas Padjadjaran, Bandung, Indonesia

**Keywords:** brazilin, anticancer agent, cytotoxic effect, iron chelation, molecular mechanism

## Abstract

Brazilin is the main compound in *Caesalpinia sappan* and *Haematoxylum braziletto*, which is identified as a homoisoflavonoid based on its molecular structure. These plants are traditionally used as an anti-inflammatory to treat fever, hemorrhage, rheumatism, skin problems, diabetes, and cardiovascular diseases. Recently, brazilin has increased its interest in cancer studies. Several findings have shown that brazilin has cytotoxic effects on colorectal cancer, breast cancer, lung cancer, multiple myeloma, osteosarcoma, cervical cancer, bladder carcinoma, also other cancers, along with numerous facts about its possible mechanisms that will be discussed. Besides its flavonoid content, brazilin is able to chelate metal ions. A study has proved that brazilin could be used as an antituberculosis agent based on its ability to chelate iron. This possible iron-chelating of brazilin and all the studies discussed in this review will lead us to the statement that, in the future, brazilin has the potency to be a chemo-preventive and anticancer agent. The article review aimed to determine the brazilin mechanism and pathogenesis of cancer.

## 1 Introduction

Cancer is the second-leading cause of death among noncommunicable diseases (NCDs), after cardiovascular disease (Cadoná et al., 2022). As of 2022, there were 19.9 million incidents of cancer overall, with 9.7 million of those cases resulting in death (IARC, 2024). According to the Global Burden of Cancer Study (GLOBOCAN), the global incidence of cancer will rapidly reach 30.2 million cases by 2040, with a mortality rate of 16.3 million cases (Sung et al., 2021). Cancer is the largest cause of death in Asia, which accounts for 49.3% of all deaths (Arnold et al., 2022).

Cancer research has always been complex due to its complexity. Despite the fact that numerous treatment options are available, their effectiveness is dependent on the stage and form of the disease. Considering the different therapeutic methods, meticulous surgical excision of aggressive tissues or tumors, chemotherapy, radiation therapy, and immunotherapy are commonly used. Surgery and radiotherapy have local effects, whereas chemotherapy and targeted therapy have systemic effects. Whether these medicines are employed individually or in combination with other treatments depends on the type and stage of cancer (Muhammad et al., 2022). They could trigger processes that promote medication resistance (Naeem et al., 2022). Combinations with additional treatments (for example, radiation therapy and conventional chemotherapy) will probably kill both normal and malignant cells, resulting in substantial hematological toxicities and tissue damage (Sharifi-Rad et al., 2021).

Historically, natural products (NPs) have played an essential role in drug discovery. Compared to conventional synthetic compounds, NPs have unique properties that provide advantages and difficulties in drug discovery. NPs are the most abundant source of high chemical diversity and structural complexity. Attempts to identify interesting therapeutic chemicals from natural sources may be one explanation for this (Feher and Schmidt, 2003; Mangal et al., 2013; Atanasov et al., 2015). Approximately 80% of NPs have previously been developed for cancer treatment (Newman and Cragg, 2016). Phenolic compounds, one of the NPS, which are molecular functional compounds that target multiple signaling pathways involved in activation or transformation of cells (Bakrim et al., 2022). Flavonoids exhibit a variety of anticancer properties, including the modification of ROS-scavenging enzyme activities, cell cycle arrest, the induction of apoptosis and autophagy, and the inhibition of cancer cell growth and invasion (Kopustinskiene et al., 2020).

*Caesalpinia sappan* is a medicinal plant with many flavonoids (Nguyen et al., 2020). *Caesalpinia sappanwood*’s high flavonoid concentration supports anti-cancer research. The primary flavonoid ingredient isolated from *C. sappanwood* is brazilin (Mottaghipisheh and Stuppner, 2021). Brazilin can also be found in heartwood trees, including brazilwood (*Caesalpinia echinata*) and bralette (*Caesalpinia Violaceae*) (Dapson and Bain, 2015). In several studies, we found that brazilin has the ability to treat several types of cancer, such as breast cancer (Jenie et al., 2018; Hermawan and Putri, 2020; Jang et al., 2020; Chatterjee et al., 2022; Haryanti et al., 2022; Yang et al., 2023), colorectal cancer (Handayani et al., 2017), multiple myeloma (Kim et al., 2012), osteosarcoma (Kang et al., 2018), lung cancer (Suyatmi et al., 2022), cervical cancer (Kitdamrongtham et al., 2013; Jeon et al., 2014), bladder carcinoma (Zhang et al., 2015; Zhang et al., 2018), and others (Lee et al., 2013; Mitani et al., 2013; Zhang et al., 2014; Bello-Martínez et al., 2017; He et al., 2017; Correia Soeiro et al., 2022; Yan et al., 2022).

A study stated that Brazilin substances can be used to chelate iron because of their structure, which reacts with metal ions such as iron (Fe) to form a stable complex (Safitri et al., 2022). Iron chelators have long been considered potential anticancer agents (Kulp et al., 1996; Kovář et al., 2001). Iron is a major substance essential in cell growth, metabolism, and replication. Metabolism of iron is regulated in cells with cancer to cope with greater replicative demands (Theil and Goss, 2009). Iron chelating substance were initially created to treat iron overload disorders, but their potential for anticancer use is becoming increasingly apparent (Ibrahim and OSullivan, 2020).

Based on the mentioned evidence, this article review aims to determine the future potential of brazilin as an anticancer agent and its mechanism to conclude whether there is a relationship between the activity of iron chelation from the brazilin compound and cancer pathogenesis.

## 2 Methods

Relevant articles were obtained from the PubMed database with the terms “brazilin” and “cancer.” In total, 32 articles were identified. We exclude 11 articles that are not in English, not review articles, not Brazilin, and not cancer studies. Therefore, the rest 21 articles were included. The relevant research articles published without any time limit were included according to the criteria.

## 3 Brazilin: Source and structure

Brazilin, one of the main compounds originating from fractionation of the heartwood extract of *C. sappan* and *Haematoxylum brasiletto*, is most widely distributed in Southeast Asia and America (Toegel et al., 2012). Traditionally, brazilin has been taken as a red dye for cosmetics, beverages, fabrics, and food in Malaysia, China, Thailand, Mexico and Vietnam because it produces a red color (Nava-Tapia et al., 2022).

Brazilin is an organic compound of the homoisoflavonoid type, named by IUPAC as (6aS,11bR)-7,11b-dihydro-6*H*-indeno [2,1-*c*]chromene-3,6a,9,10-tetrol, with molecular formula C_16_H_14_O_5_ and the molecular weight 286.28 g/mol (National Center for Biotechnology Information, 2023). It may also be named as natural red, braziletto, or brasilin (Edwards et al., 2003). Brazilin is a colorless phenolic compound consisting of one pyrone, one five-membered ring, and two aromatic rings (Rondão et al., 2013). However, the hydroxyl group of the brazilin structure is readily oxidized. It can be converted into carbonyl groups, leading to transformation of the structure and forming a colored substance called Brazilein (Harborne et al., 2013).

Brazilin is the main component of the crude dye, and brazilein is a polyphenolic compound that may be separated in large amounts by exposing the organic extract to air and light, which oxidizes the hydroxyl of brazilin to a carbonyl group. Brazilin and brazilein are tetracyclic. The aromatic ring attached to the pyrone ring should originate in the acetate pathway, while the aromatic ring bonded to the five-membered ring in the shikimic acid pathway (de Oliveira et al., 2002).

## 4 Brazilin pharmacological activities

Traditionally, extracts of *C. sappan* and *H. brasiletto* have been applied to fever, hemorrhage, diabetes, skin problems, cardiovascular diseases, and diabetes as an anti-inflammatory because of their potential for medicinal use (Pawar et al., 2008; Nirmal and Panichayupakaranant, 2015; Mueller et al., 2016; Hwang and Shim, 2018). Specifically, as a major compound of both plants, brazilin possesses various pharmacological activities. Brazilin is a significant inhibitor of nitrite oxide (NO) production. It is a valuable therapy for antioxidants, anti-inflammation, and vascular relaxation (Hu et al., 2003; Sasaki et al., 2007). Brazilin significantly inhibited J444.1 cell line nitrite oxide (NO) generation produced by lipopolysaccharide (LPS). It has been discovered that brazilin significantly lowers iNOS gene expression at 100 μM, while its derivative, brazilein, does so even at 10 µM^54^. As an essential relaxing factor in the circulatory system, brazilin increases NO production, NOS activity, and extracellular Ca^2+^ influx in human umbilical vein endothelial cells (Hu et al., 2003). Compared to its derivate, Brazilein, which has activity to reducing liver damage that caused by excess iron, increasing cytotoxicity and apoptosis in T47D cells, and inhibiting NFκB1/p50 in human osteoarthritic (Safitri et al., 2016; Utomo et al., 2018; Weinmann et al., 2018). Brazilein also can be invented as potential antibacterial agent against *Escherichia coli* MDR, a sensitive antibiotic using *in silico* method (Krihariyani et al., 2020).

Brazilin has antimicrobial effects through decreasing DNA and protein production. Brazilin inhibited the growth of bacteria that cause methicillin-resistant *Staphylococcus aureus* (MRSA), dental caries (*Streptococcus mutans*), periodontal disease (*Prevotella intermedia*), acne (*Propionibacterium acnes*), and strep throat (Group A strep) (Xu and Lee, 2004). Brazilin has an increased pyruvate kinase activity mechanism. In addition, it may play a role in the anti-gluconeogenic action of brazilin. Brazilin enhanced the levels of 6-phosphofructo-2-kinase (PFK-2), fructose-6-phosphate (F-6-P), and hexose-6-phosphate (H-6-P) extensively (You et al., 2005).

## 5 Brazilin mechanisms in cancer

Brazilin has been studied in several types of cancer, such as cervical cancer, cervical squamous cell carcinoma, breast cancer, colorectal adenocarcinoma, colorectal cancer, colon cancer, hepatocellular carcinoma, lung adenocarcinoma, and sarcoma. A compound’s ability to impede biological or biochemical function may be measured through its half-maximal inhibitory concentration (IC_50_) (Hendriks, 2010). Drug potency, or the quantity of a drug required to have a therapeutic effect, is correlated with the IC_50_ value. Like a drug, the IC_50_ value of a natural compound must be determined to know the cytotoxicity. The lower the IC_50_ value, the more cytotoxic the substance is (Meyer et al., 2019). Based on its IC_50_ value from different types of cancer cell lines (Bello-Martínez et al., 2017; Handayani et al., 2017; Haryanti et al., 2022; Jenie et al., 2018; Suyatmi et al., 2022; Yang et al., 2023; Zhang et al., 2014), brazilin has a strong ability to treat breast cancer on 4T1 cell lines with the measured IC_50_ value of 3.7 µM. The methods used in several studies to know the mechanism of brazilin to treat several types of cancer are shown in Table 1.

**TABLE 1:** Summary of Brazilin activity in several types of cancer.

No.	Methods			Mechanism	References
	*In Silico*	*In Vitro*	*In Vivo*		
1	—	Breast cancer MDA-MB-231 and 4T1 cells	Subcutaneous xenotransplantation in BALB/C mice	^-^	Yang et al. (2023)
2	*MMP14*, *PTGS2*, *ADAM17*, *PTEN*, *CCL2*, *PIK3CB*, *MAP3K8*, and *CXCL3*	-	-	TNFα signaling	Hermawan and Putri (2020)
3	-	Breast cancer MCF-7/HER-2 cells	-	Cytotoxicity and cell migration	Jenie et al. (2018)
4	MMP-9, MMP-2, and PTGS2 enzymes	Triple Negative Breast Cancer (TNBC) 4T1 cell line	-	Cell migration	Haryanti et al. (2022)
5	-	Human breast cancer MCF-7 cell line		Hemin-induced HO-1 protein expression	Jang et al. (2020)
6	S-adenosyl-L-homocysteine (SAH) and DNMT1 protein	Human breast cancer cell line MCF7 and the gene expression of DNMT1, p38 MAPK, p53, and p21		Cell proliferation and DNMT1 expression	Chatterjee et al. (2022)
7	-	Colorectal cancer WiDr cell line		Apoptosis and Cell cycle	Handayani et al. (2017)
8	-	Multiple Myeloma U266 cell line	-	Histone deacetylases (HDACs)	Kim et al. (2012)
9	-	Osteosarcoma MG-63 cell line	-	Apoptosis	Kang et al. (2018)
10	-	Non-Small Lung Carcinoma A549 cell line	-	Intrinsic apoptosis	Suyatmi et al. (2022)
11	-	Human cervical cancer HeLa cell line	-	NF-κB luciferase	Jeon et al. (2014)
12	-	SRB assay in human cervical cancer HeLa cell line	HeLa xenograft and sub chronic toxicity in nude mice and rats	Antitumor and antiproliferative	Kitdamrongtham et al. (2013)
13	-	Bladder carcinoma T24 cell line	-	Cell proliferation	T. Zhang et al. (2015)
14	-	Bladder carcinoma T24 cell line	-	Apoptosis	T. Zhang et al. (2018)
15	BAF1 (barrier-to-autointegration factor 1) protein	-	-	-	Correia Soeiro et al. (2022)
16	-	Glioblastoma multiforme U87 cell line	-	Cell growth and apoptosis	Lee et al. (2013)
17	-	Head and neck squamous cell carcinoma	-	Apoptosis	He et al. (2017)
18	-	SiHa, MDA-MB-231, A549, and NCI-H1299 cell lines	-	Cell proliferation	Bello-Martínez et al. (2017)
19	-	HEK293T cell line and cancer cell lines, including HTC75, HeLa, DLD1, MDA-MB-231, Hs578t and A549	6–8-week-old nude mice *in situ*	Telomerase *in vitro* and *in vivo*	Yan et al. (2022)
20	-	Human melanoma HMV-II cell line	-	Tyrosinase and *TYRP2* mRNAs	Mitani et al. (2013)
21	-	RAW 264.7 macrophage cells	S180 mouse sarcoma cells	DNA binding activity of NF-κB and AP-1	Zhang et al. (2014)

In treating cancer, brazilin has different types of mechanisms, but the general mechanism is to induce apoptosis and inhibit cell proliferation. Most of the studies were carried out using breast cancer cell lines. Brazilin inhibits cell proliferation, migration, and invasion as the primary therapy and co-therapy with doxorubicin (Jenie et al., 2018; Haryanti et al., 2022). Hemin-induced heme oxygenase-1 (HO-1) in breast cancer cells is slightly inhibited by brazilin (Jang et al., 2020). Besides, HO-1 has cytoprotective properties to promote cancer progression in cancer cells, yet HO-1 overactivation also promotes unconventional ferroptosis due to an accumulation of prooxidant-free iron (Nitti et al., 2021). Other findings were obtained to help arrange possible molecular mechanisms of brazilin as a novel anticancer agent. Brazilin suppressed the activity of transcription factors called histone deacetylases (HDACs), which are involved in controlling cell cycle arrest and apoptosis in multiple myeloma (Kim et al., 2012).

An *in silico* study predicted several targets that brazilin inhibited. In triple breast cancer, brazilin was found to attach more firmly and effectively to MMP-2, MMP-9, and PTGS2 compared to its native ligand (Haryanti et al., 2022). These enzymes are involved in cell migration and metastasis (Webb et al., 2017; Ercolano et al., 2019). According to these findings, brazilin could potentially disrupt the activities of the enzymes by binding to their active sites (Haryanti et al., 2022).

Brazilin has also shown its activity in several other types of cancer, but its mechanism has not yet been studied further. Brazilin mechanisms are limited to *in silico*, *in vitro*, and *in vivo*. No clinical research has ever been done. Apoptotic-related pathways are still the most interesting to explore for a new potential anticancer agent. Every discovery is valuable to guide us for new pathways regarding anticancer therapy mechanisms by brazilin. Of all the findings about brazilin mechanisms towards cancer, the iron chelation mechanism has not been mentioned yet. In the meantime, using iron chelators as an adjuvant cancer treatment is becoming more popular, even though they have been developed initially to treat iron overload disorders (Wang et al., 2019; Safitri et al., 2022).

## 6 Brazilin as iron chelators

Brazilin belongs to the class of flavonoids that are likely to interact with metals, mainly Fe (Kejík et al., 2021). A study has found that brazilin could be used as an antituberculosis because of its mechanism of inhibiting *Mycobacterium tuberculosis* (Mtb) extracellularly by iron chelation (Safitri et al., 2022). Iron overload removal and prevention are the main goals of chelation therapy. By chelating extra iron, iron levels can be maintained at normal levels. Iron chelation therapy was first developed for transfusion-dependent anemias, including myelodysplasia, sickle cell disease, and thalassemia. However, this is only a small portion of the potential spectrum of activity for iron chelators (Porter, 2001). Transferrin (Tf), a protein with a strong affinity for iron, carries iron in plasma. After the iron transferrin complex attaches to the cell surface’s transferrin receptor 1 (TfR1), the complex is internalized through receptor-mediated endocytosis, and endosomal acidification releases the iron from Tf (Hentze et al., 2004). On the other hand, cancer cells have a few different mechanisms to keep the balance of iron within their cells. In neoplastic cells, iron metabolism is altered in order to fulfill higher replicative needs. Numerous processes contribute to the increased iron uptake in neoplastic cells, but the most prominent one is the increased protein expression of the TfR1 receptor, which has been found in several cases, including renal, colorectal, liver, breast, and lung cancer (Kindrat et al., 2016; Greene et al., 2017; Horniblow et al., 2017; Rychtarcikova et al., 2017). In these neoplasms, its level has been connected to the growth of the tumor (Brookes et al., 2006). Numerous neoplasms have also been reported to have elevated levels of the homologous TfR2 (Calzolari et al., 2007). It has been shown in melanoma and hepatoma cell lines that intake occurs via non-receptor-mediated pinocytosis once TfR1 is saturated (Richardson and Baker, 1994; Trinder et al., 1996).

Iron chelators that are clinically approved are as follows: Deferoxamine (DFO) (Kontoghiorghes et al., 1987; Hernlem et al., 1996), Deferiprone (L1) (Rombos et al., 2000; Cohen et al., 2003; Hoffbrand et al., 2003), and Deferasirox (DFX) (Gaboriau et al., 2010). Iron chelators were initially developed to treat iron overload disorders like thalassemia, but there is increasing interest in their potential as adjunctive therapy for cancer. The combination of iron-chelating agents like DFO or DFX with cisplatin, doxorubicin, and carboplatin has been shown to increase the cytotoxic effects of these chemotherapeutics in some studies (Wang et al., 2019; Safitri et al., 2022). Another natural compound that has iron chelator activity is curcumin. Curcumin shows great potential as a therapeutic substance and is being studied in humans for a number of diseases, such as psoriasis, pancreatic cancer, multiple myeloma, colon cancer, and myelodysplastic syndromes. Curcumin inhibits the development and progression of cancer by targeting different stages in the malignant process (Hatcher et al., 2009).

**FIGURE 1:**
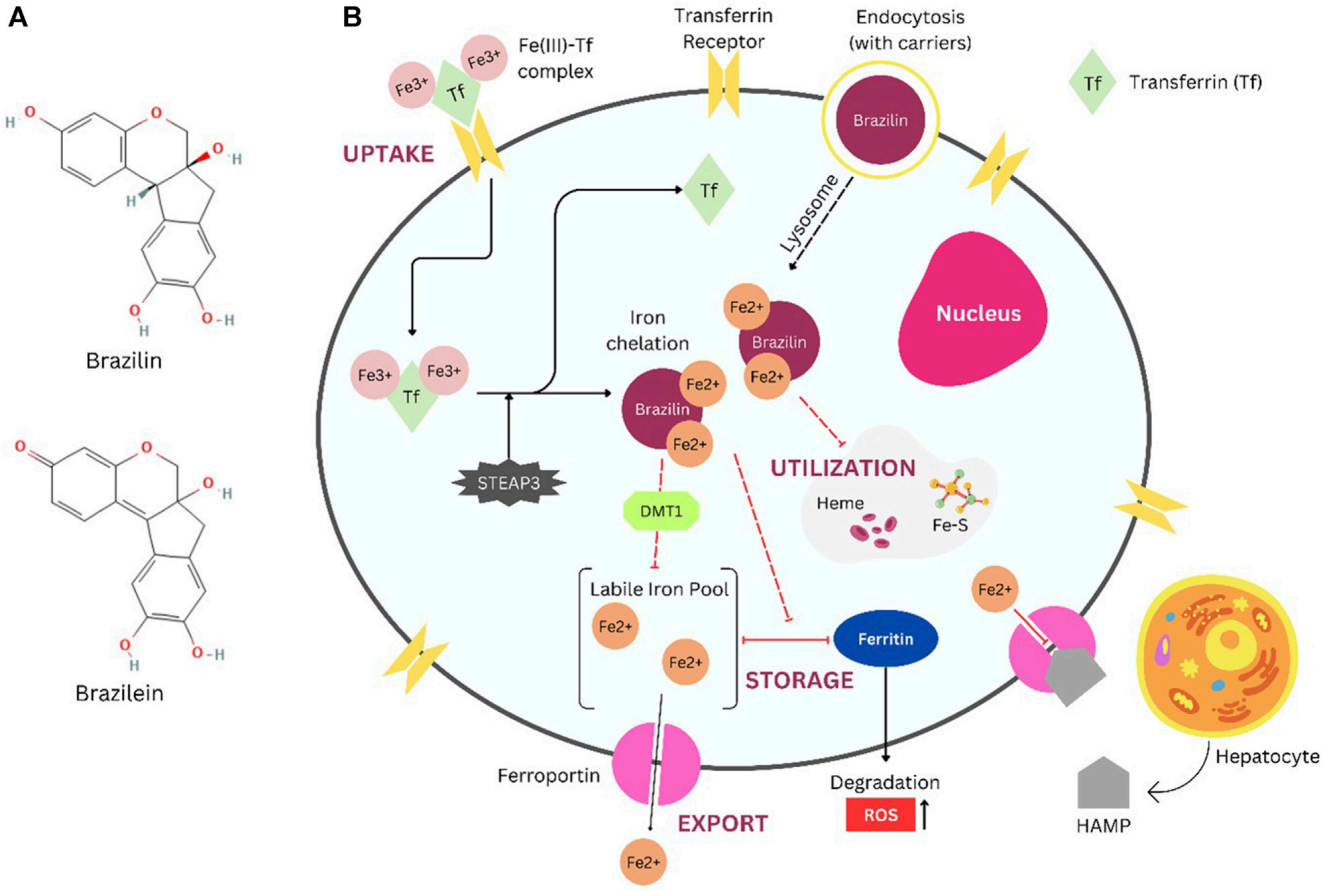
Brazilin (National Center for Biotechnology Information, 2024b) and Brazilein ((National Center for Biotechnology Information, 2024a) structure **(A)**, Brazilin predicted mechanism as iron chelators in cancer cells **(B)**. Illustration refers to the common mechanism of clinically approved iron chelators (i.e., Deferoxamine (DFO)). DFO and other compounds with similar structures have poor pharmacokinetics profiles. Conjugation with drug carriers, such as polymers, could help increase therapeutical efficacy (Komoto et al., 2021). Brazilin with carriers (polymer, plasmid, etc.) entering cell cytoplasm via endocytosis. Iron uptake involves Transferrin that binds two irons and enters cells by interaction with the Transferrin Receptor (TfR1). Iron is typically transported by DMT1 toward the labile iron pool (LIP) and then scavenged and stored by Ferritin (Pfeifhofer-Obermair et al., 2018). In cancer cells, the requirements of iron are relatively high, and there is also an accumulation of free iron inside cells caused by Hepcidin (HAMP) binding to Ferroportin (FPN), which is beneficial for iron export to maintain iron homeostasis (Bystrom et al., 2014). Iron chelation occurs inside cells toward free labile iron (Fe^2+^). Thereby, storage and utilization of iron are inhibited by this chelation mechanism. Further research is recommended to clarify whether brazilin promotes pro-oxidant and iron depletion effects. **Abbreviations:** DMT1, divalent metal transporter 1; Fe-S, iron sulfide; HAMP, hepcidin antimicrobial peptide; ROS, reactive oxygen species; STEAP3, six-transmembrane epithelial antigen of prostate 3; Tf, transferrin.

Due to the bidentate ligand, a strong metal cation scavenger that can tightly bind iron (III) at pH 7, brazilin also happens to have a catechol group that may chelate iron, according to various research (Hruby et al., 2021). Brazilin’s structure shares a few similarities to that of DFO, a hexadentate compound that can bind iron in a 1:1 ratio to form a stable complex that prevents the free radicals that iron produces (Zhou et al., 2012). An iron chelator’s potency is determined by how well it can bind transferrin-bound iron that is not circulating in the plasma. Among other iron chelators, L1 is considered very effective in iron chelating (Maskoen et al., 2016). The substance has the benefit of quickly penetrating membranes to remove potentially harmful iron from tissues since the Fe(III) chelate of L1 has no net charge (Kattamis et al., 2006). To chelate one iron atom, L1 molecules are required (Kontoghiorghes et al., 1987; Merson and Olivier, 2002). Structurally, brazilin has properties similar to L1 due to its bidentate structure, indicating that brazilin can bind iron with the same ratio as L1. Mechanism prediction of brazilin as iron chelators is shown in Figure 1.

## 7 Iron chelating activity in cancer pathogenesis

The development of iron chelators as therapeutic agents can also be useful anticancer agents (Buss et al., 2005) either by depleting iron in the tumor or by causing selective oxidative stress in the tumor due to redox perturbations in its environment (Hatcher et al., 2009; Fibach and Rachmilewitz, 2017). One of the metabolic characteristics of malignant cancer cells is dysregulated iron homeostasis, where iron is crucial for the growth, survival, proliferation, and metastasis of tumors at every stage of the process (Ludwig et al., 2015). Tumor cells are more susceptible to iron deficiency than normal cells because they rely heavily on iron for development and proliferation (Bystrom and Rivella, 2015). Iron reductase, primarily found in some members of the metal reductases six-transmembrane epithelial antigen of the prostate (STEAP1-4) family, reduces Fe^3+^ to Fe^2+^ in the endosome. Many human cancer types, including breast, colon, prostate, cervix, bladder, pancreatic, testis, ovary and Ewing sarcoma, have high expression levels of STEAP1 and STEAP2. In malignant gliomas, STEAP3 is overexpressed, and STEAP3 knockdown suppresses glioma cell metastasis, proliferation, and clonality, *in vitro* and tumor growth *in vivo*. Under hypoxic conditions, STEAP4 is activated, which increases the incidence of colitis-associated colon cancer in mice models, enhances the formation of reactive oxygen species (ROS), and causes an iron imbalance in the mitochondria (Wang et al., 2019).

Deferoxamine (DFO) and Deferasirox (DFX) are widely used for iron overload disease in cancer therapy (Ibrahim and OSullivan, 2020). Among all iron chelators available on market, DFX is the first-choice iron chelator used globally to treat non-transfusion-dependent thalassemia syndromes in patients from age of 10 and above, along with chronic iron overload on by blood transfusions in patients from age of 2 (Piga et al., 2006). DFO chelates non-transferrin bound iron (free iron), hemosiderin, and iron in transit between transferrin and ferritin (labile chelating iron pool). DFO is able to directly attach to iron and remove it away from heart cells, but it is unable to bind iron that has already been integrated into other molecules, such as hemoglobin, transferrin, or cytochromes (Hershko et al., 2001; Komoto et al., 2021). However, DFX preferentially binds to iron in its oxidized ferric (Fe^3+^) state than to the reduced or ferrous (Fe^2+^) state. Every DFX molecule binds two ferric irons (Valentovic and Enna, 2008). Additionally, it was discovered that DFX caused apoptosis by reducing ER stress responses (Kim et al., 2016). Based on previous discussion, we conclude that brazilin has the potential ability to chelate iron. Concerning its activity, it has previously been developed as a targeted anticancer therapy. As a result, brazilin can potentially be an anticancer agent through the iron chelation mechanism.

## 8 Discussion

Brazilin has different mechanisms in treating cancer, but mainly induces apoptosis and inhibits cell proliferation. Most of the studies were carried out using breast cancer cell lines. Brazilin inhibits cell proliferation, migration, and invasion as the primary therapy and co-therapy with doxorubicin (Jenie et al., 2018; Haryanti et al., 2022). Brazilian mechanism studies are limited to *in silico* and *in vivo.* Every discovery is valuable to guide us for new pathways regarding anticancer therapy mechanisms by brazilin. The iron chelation mechanism in brazilin to treat cancer is not mentioned yet. Iron chelators were originally created to treat iron overload problems, however there has been rising interest in using them as adjuvant therapy for cancer (Wang et al., 2019; Safitri et al., 2022).

Other pathways are present in cancer cells for keeping the equilibrium of iron within the cell. In neoplastic cells, iron metabolism is altered to satisfy higher replicative needs. Numerous processes contribute to the increased iron uptake in neoplastic cells, but the most prominent one is the increased protein expression of the TfR1 receptor, which has been found in several cases, including renal, colorectal, liver, breast, and lung cancer (Kindrat et al., 2016; Greene et al., 2017; Horniblow et al., 2017; Rychtarcikova et al., 2017). Brazilin’s bidentate ligand, a strong metal cation scavenger that can tightly bind iron (III) at pH 7, is responsible for the catechol group’s ability to chelate iron (Hruby et al., 2021). Brazilin’s structure shares a few similarities to that of deferoxamine (DFO), a hexadentate compound that can bind iron in a 1:1 ratio to form a stable complex that prevents the free radicals that iron produces (Zhou et al., 2012).

In conclusion, we have found that brazilin has activities toward cancer pathogenesis via apoptosis mechanism and cell cycle arrest. There is a study stating that brazilin has the ability to chelate iron as an antituberculosis agent. The development of iron chelators also can be useful as anticancer agents. Throughout the whole process of tumor growth, survival, proliferation, and metastasis, iron is essential. Therefore, brazilin has the potential as an anticancer agent through the iron chelation mechanism. However, further research and investigations must be conducted on brazilin to confirm this finding.
